# Multi-epitope protein production and its application in the diagnosis of opisthorchiasis

**DOI:** 10.1186/s13071-024-06285-7

**Published:** 2024-05-07

**Authors:** Jittiyawadee Sripa, Tarinee Chaiwong

**Affiliations:** 1 College of Medicine and Public Health, Ubon Ratchathani University, Warinchamrap, 34190 Ubon Ratchathani Thailand; 2 Research Group for Biomedical Research and Innovative Development (RG-BRID), College of Medicine and Public Health, Ubon Ratchathani University, Warinchamrap, 34190 Ubon Ratchathani Thailand

**Keywords:** Multi-antigenic protein, B cell epitopes, Immunoblotting, Opisthorchiasis

## Abstract

**Background:**

Opisthorchiasis and cholangiocarcinoma (CCA) continue to be public health concerns in many Southeast Asian countries. Although the prevalence of opisthorchiasis is declining, reported cases tend to have a light-intensity infection. Therefore, early detection by using sensitive methods is necessary. Several sensitive methods have been developed to detect opisthorchiasis. The immunological detection of antigenic proteins has been proposed as a sensitive method for examining opisthorchiasis.

**Methods:**

The *Opisthorchis viverrini* antigenic proteins, including cathepsin B (OvCB), asparaginyl endopeptidase (OvAEP), and cathepsin F (OvCF), were used to construct multi-antigenic proteins. The protein sequences of OvCB, OvAEP, and OvCF, with a high probability of B cell epitopes, were selected using BepiPred 1.0 and the IEDB Analysis Resource. These protein fragments were combined to form OvCB_OvAEP_OvCF recombinant DNA, which was then used to produce a recombinant protein in *Escherichia coli* strain BL21(DE3). The potency of the recombinant protein as a diagnostic target for opisthorchiasis was assessed using immunoblotting and compared with that of the gold standard method, the modified formalin-ether concentration technique.

**Results:**

The recombinant OvCB_OvAEP_OvCF protein showed strong reactivity with total immunoglobulin G (IgG) antibodies against light-intensity *O. viverrini* infections in the endemic areas. Consequently, a high sensitivity (100%) for diagnosing opisthorchiasis was reported. However, cross-reactivity with sera from other helminth and protozoan infections (including taeniasis, strongyloidiasis, giardiasis, *E. coli* infection, enterobiasis, and mixed infection of *Echinostome* spp. and *Taenia* spp.) and no reactivity with sera from patients with non-parasitic infections led to a reduced specificity of 78.4%. In addition, the false negative rate (FNR), false positive rate (FPR), positive predictive value (PPV), negative predictive value (NPV), and diagnostic accuracy were 0%, 21.6%, 81.4%, 100%, and 88.9%, respectively.

**Conclusions:**

The high sensitivity of the recombinant OvCB_OvAEP_OvCF protein in detecting opisthorchiasis demonstrates its potential as an opisthorchiasis screening target. Nonetheless, research on reducing cross-reactivity should be undertaken by detecting other antibodies in other sample types, such as saliva, urine, and feces.

**Graphical Abstract:**

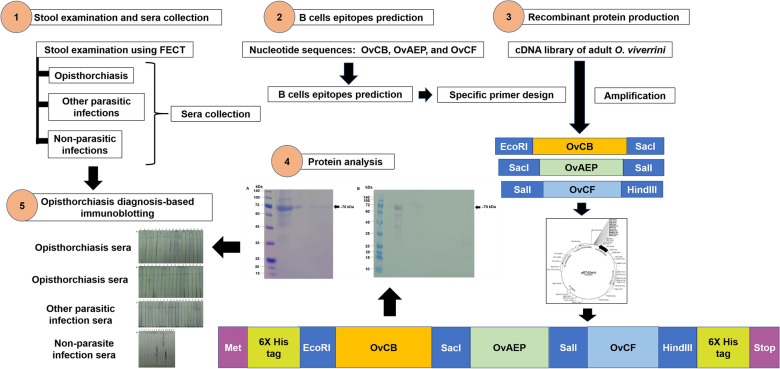

**Supplementary Information:**

The online version contains supplementary material available at 10.1186/s13071-024-06285-7.

## Background

Opisthorchiasis is a major public health concern in several Southeast Asian countries. The prevalence of opisthorchiasis has decreased in many regions because of intensive prevention and control programs, mass stool examinations, and drug treatments; however, most reported cases have shifted to light infections [1–4]. Thus, a sensitive and specific examination is required for the early diagnosis of opisthorchiasis to ensure early chemotherapy and behavioral modifications in patients.

Molecular and immunological techniques have been investigated as alternative methods to overcome the limitations of traditional microscopic stool examinations, including low sensitivity [5], mistaking small intestinal flukes [6], and the inconvenience of fecal collection, which has prompted several residents to deny involvement in stool examinations [7–9].

Identifying strong reactivity between antigenic antigens and specific antibodies related to *Opisthorchis viverrini* infection is beneficial for diagnosing opisthorchiasis. Several antigenic antigens related to *O. viverrini*, such as snail antigens [10–13], *O. viverrini* excretory/secretory products (ES), crude antigens, and other secreted proteins, have been examined as diagnostic targets. Among the well-established antigens, secreted proteins such as cathepsin B (OvCB) [14], cathepsin F (OvCF) [15], and asparaginyl endopeptidase (OvAEP) [16] have been detected in ES and crude somatic extracts of *O. viverrini*. Thus, the host immune system can be exposed to OvCB, OvAEP, or OvCF to induce a humoral immune response.

The potential of OvCB, OvAEP, and OvCF to induce humoral immune responses and their high immunogenicity have been demonstrated by the strong reactivity between recombinant proteins and specific antibodies against *O. viverrini* infection. This strong immunological reaction indicated the presence of B-cell epitopes in the protein sequences of OvCB, OvAEP, and OvCF. Thus, modifying a single protein by combining it with OvCB, OvAEP, and OvCF protein fragments that represent a high epitope to B cells could increase the immunogenicity of the protein target and the sensitivity of immunological detection methods.

Thus, in this study, protein sequences from OvCB, OvAEP, and OvCF, which represent high-probability of B-cell epitopes, were selected to construct a multi-epitope recombinant protein. Subsequently, the efficacy of the modified recombinant protein in the diagnosis of opisthorchiasis was tested. Human negative and positive parasitic infection sera, including opisthorchiasis and other helminth and protozoan infections, were used to test and establish immunoblotting. The sensitivity and specificity of the test were evaluated and compared with those of the gold standard method, the modified formalin–ether concentration technique (m-FECT).

## Methods

### Human sera

The parasitic infection sera used in this study were obtained from individuals with helminth eggs, larvae, and protozoan cysts detected in their feces using m-FECT. The individual parasitic infections were opisthorchiasis (35 cases), taeniasis (4 cases), strongyloidiasis (5 cases), hookworm infection (3 cases), giardiasis (3 cases), *Entamoeba coli* infection (2 cases), enterobiasis (1 case), trichuriasis (1 case), and mixed infections with *Echinostome* spp. and *Taenia* spp. (1 case). Individual negative sera (17 sera) obtained from subjects who lived in endemic areas and were negative for parasitic infection with m-FECT were included to examine the specificity and accuracy of the diagnosis. The intensity of helminth infections, including opisthorchiasis, hookworm infection, trichuriasis, and echinostomiasis, was low. All procedures involving human participants were performed in accordance with the Declaration of Helsinki. The procedures used in this study were reviewed and approved by the Human Ethics Committee of Ubon Ratchathani University (UBU-REC-46-2563).

### B-cell epitopes prediction

The amino acid sequences of *O. viverrini* cathepsin B; OvCB (GenBank accession no. GQ303559.1), asparaginyl endopeptidase, or legumain; OvAEP (GenBank accession no. DQ402101.1) and cathepsin F or cysteine protease; and OvCF (GenBank accession no. AY821800.1), retrieved from the NCBI database, were used to predict linear B-cell epitopes using BepiPred 1.0 and the IEDB Analysis Resource. The peptide fragments of each protein showed epitope probability scores for B cells, and the peptide fragments with the highest scores were selected [17].

### Construction of multi-epitopes recombinant DNA

The DNA sequences corresponding to the selected peptide fragments were used as templates for primer design. Primer pairs were designed to encompass the selected OvCB, OvAEP, and OvCF DNA sequences. Each primer was incorporated with restriction sites to facilitate fusion with other selected DNA sequences and ligation into pET32a+ (Table 1).

**Table 1:** Selected DNA sequences of OvCB, OvAEP, and OvCF encoded high B-cell epitope probability peptides

Genes	The B-cell epitope-rich peptides	The selected cDNA encoded B-cell epitope	B-cell epitope probability	Primers	Total length of DNA (bp)
Cathepsin B (GenBank accession no. GQ303559.1)	21–40, 48–87, 89–108, 132–139, 150–157, 168–227, 238–240, 242, 263–279, 307–310	**GAA TTC** ACT GGA GCA CGA TGG ATA TCT GGA AGA CAT TCG AAA GGA TTC GAA TCT GAC CAC CTG ATT CAC ACG TTT GGA GCC AAG ATG GAA ACT GCA GAA CAA AAA GCG CAG AGG CCA ACG GTC AAG CAC GTG GGT TTT GAT GAT ACG CGT CTC CCA AAG AAC TTT GAT GCA CGA TCT AAA TGG CCG CAT TGC TCT TCC GTC AGT GAG ATC AGA GAT CAA TCC AGT TGT GGA TCG TGT TGG GCG TTC GGG GCA GTG GAA GCC ATG AGT GAT CGA CTG TGC ATT CAT TCA AAT GGT TCT TTC AAC AAA AGC CTC AGT GCG GTA GAC TTG CTC TCC TGT TGT AAG GAC TGT GGA TTC GGT TGT CGT GGA GGA TAT CCT GCT GTG GCG TGG GAC TAC TGG AGG ACT CAC GGC ATT GTC ACA GGT GGT TCA AAA GAA GAT CCA AGT GGA TGC AGG TCT TAT CCA TTT CCG AAA TGT GAC CAT CAT GTT CAA GGA CAC TAT CCG CCA TGT CCG CGT CAA ATC TAC CCC ACA CCG GAA TGC GTC CAG GAC TGT GAC ACG CCA GAA TTG GGT TAC TTG GAG GAT AAG ACG AGA GCT AAC ATC TCC **GAG CTC**	0.493	Fwd: 5'- GCG CGC **GAA TTC** ACT GGA GCA CGA TGG ATA TCT GGA -3' Rev: 5'- GCG CGC **GAG CTC** GGA GAT GTT AGC TCT CGT CTT ATC -3'	603
Asparaginyl endopeptidase, also known as legumain (GenBank accession no. DQ402101.1)	24–34, 68–70, 85–91, 97–119, 127–141, 202–206, 220–231, 249–260, 265–294, 304–317, 321, 323, 339–357, 370–378, 393–401	**GAG CTC** GAG CAT CAC GAT CTG TCG CAT CGC ACA CTG GAT GAT CAG TTC CAA TCG GTG AAA CAG AAT ACC AAG CAA AGT CAC GTA TCG AGA TTC GGG GAA CTG CCT CAG GTA CTT CAT AGC CAT CCG TCA CGC TGG GCA CAT TTG GTC ACC ATG GTC CGA CGA ATG ATG AAA GCC GAA ACC GAG GAA GAA CAT GAA TTG GCA TCC CGA AAA CTA TAT CGT GCA CTT CTG CTT GCC CAG ATC GTT AAA GAA ACA TTC GAA GAA ATC GTC ACG GAT GTA ACA ACC TTC CAT CAG CCA ACC ATG CGC ATG TTG TCA AAG TCG GAG GAA CTC CAG **GTC GAC**	0.469	Fwd: 5'- GCG CGC **GAG CTC** GAG CAT CAC GAT CTG TCG CAT CGC -3' Rev: 5'- GCG CGC **GTC GAC** CTG GAG TTC CTC CGA CTT TGA CAA -3'	336
Cathepsin F (GenBank accession no. AY821800.1)	22–31, 37–53, 60–75, 78–133, 155–163, 174–177, 192–219, 225–234, 254–269, 283–286, 298–300	**GTC GAC** CAA TTT TCC GAC CTG ACC AGT GAG GAG TTC AAG ACG CGG TAT TTG AGG ATG CGA TTT GAT GAG CCG ATT GTC AAT GAG GAT CCC ACC CCA CAA GAA GAT GTG ACG ATG GAT AAC AGC AAT TTT GAT TGG CGA GAT CAT GGT GCA GTC GGA CCA GTA TTG GAC CAA GGA GAT TGT GGT TCG TGC TGG GCA TTT TCT GTG ATT GGG AAT GTC GAG GGT CAG TGG TTC CGT AAG ACT GGG GAT CTA CTA GGT **AAG CTT**	0.482	Fwd: 5'- GCG CGC **GTC GAC** CAA TTT TCC GAC CTG ACC AGT GAG -3' Rev: 5'- GCG CGC **AAG CTT** ACC TAG TAG ATC CCC AGT CTT ACG -3'	261

The selected nucleotide sequence included the forward and reverse primers priming sites (underlined). The restriction sites were incorporated in the specific primers (bold and underlined)

The selected DNA segments of OvCB, OvAEP, and OvCF amplified in 25 μL of polymerase chain reaction (PCR) mixture containing 10 mM dNTP, 1X SuperFi™ II buffer, 0.5 μM of each primer, 1 μL of Platinum SuperFi II DNA Polymerase (Thermofisher Scientific), 200 ng of cDNA library of adult *O. viverrini*, and distilled water were added to bring the mixture’s volume up to 25 μL. The PCR reactions were subjected to amplification in Biometra T-Personal 48 Thermocycler (Analytik Jena GmbH, Germany) with the conditions as following: pre-denaturation at 95 °C for 5 min, followed by 35 cycles of denaturation at 95 °C for 1 min, annealing at 60 °C for 45 s, extension at 72 °C for 1.5 min, and a final extension at 72 °C for 10 min.

The PCR products were verified using 1% agarose gel electrophoresis, and the lengths of DNA fragments were estimated by comparison with VC 100 bp Plus DNA Ladder (Vivantis Technologies, Malaysia). The DNA fragments were purified from the agarose gel using the GeneJET Gel Extraction Kit (Thermofisher Scientific). The purified PCR products were digested with the respective restriction enzymes (Thermofisher Scientific) at 37 °C for 3–5 h. Then, the digested PCR products were dissolved on 1% agarose gel electrophoresis, and DNA was purified from agarose gel using GeneJET Gel Extraction Kit (Thermofisher Scientific).

The pET32a + vector was digested with the respective restriction enzymes before ligation with the selected DNA fragments of OvCB, OvAEP, and OvCF. Briefly, the pET32a+ vector in *Escherichia coli* strain TOP10 was propagated overnight in Luria–Bertani (LB) broth at 37 °C in a shaking incubator at 200 rpm. Then, the bacterial pellet was collected and extracted for pET32a+ vector using PureLink™ Quick Plasmid Miniprep Kit (Invitrogen™, Thermofisher Scientific). The pET32a+ vector was digested with the corresponding restriction enzymes with the DNA fragments of OvCB, OvAEP, and OvCF, respectively. The digested vector was verified by 1% agarose gel electrophoresis and the linearized vector was purified from agarose gel using GeneJET Gel Extraction Kit (Thermofisher Scientific).

**Fig. 1:**
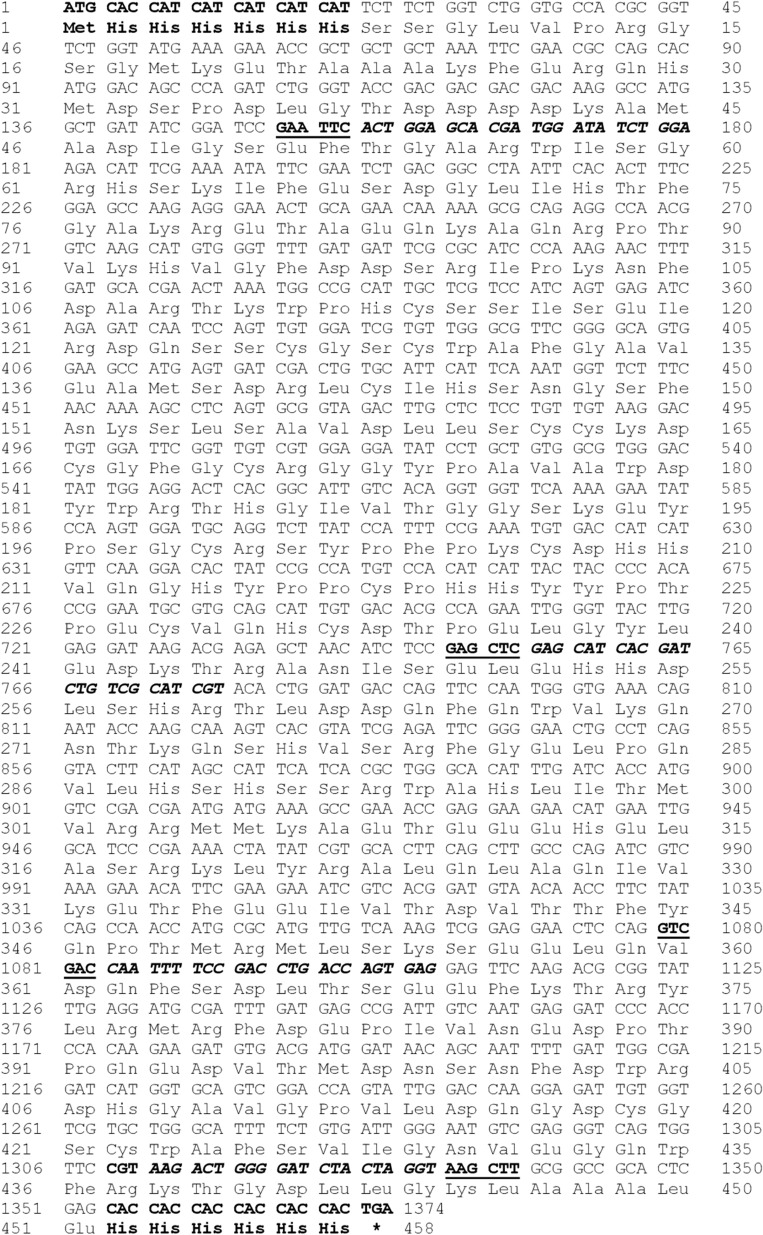
The deduced amino acids sequence of recombinant DNA of OvCB_OvAEP_OvCF in pET32a+ . The start codon, Met, and the stop codon (*) of the vector were highlighted. The 6 × His sequence tags were shown in both N- and C-terminals of the peptide (bold and underlined were restriction sites, GAA TCC: EcoR I; GAG CTC: SacI; GTC GAC: SalI and AAG CTT: HindIII, bold and italics were primer priming sites)

The OvCB, OvAEP, and OvCF DNA fragments were ligated into the linearized pET32a+ vector (Fig. 1) using T4 ligase (Thermofisher Scientific) following the manufacturer’s protocol. The ligation products confirmed the integration of DNA fragments with a pair of T7 sequencing primers before being transformed into *E. coli* competent cells (strain TOP10) using a chemical transformation method. Positively transformed colonies were selected on LB agar containing 100 μg/mL of ampicillin. Colony PCR was performed with a set of T7 sequencing primers and specific primers to verify integration of the DNA fragments. The plasmid DNA clones were subsequently sequenced, and the results were analyzed using BioEdit version 7.2.

The recombinant DNA OvCB_OvAEP_OvCF_pET32a+ was constructed. DNA fragments of OvAEP and OvCF were cleaved from recombinant OvAEP_pET32a+ and OvCF_pET32a+ DNA, respectively. The DNA fragments OvAEP and OvCF were ligated into the recombinant DNA, OvCB_pET32a+, to construct OvCB_OvAEP_OvCF_pET32a+ (Fig. 1). Then, the ligated OvCB_OvAEP_OvCF_pET32a+ was transformed into a bacterial expression host, *E. coli* strain BL21(DE3), using the chemical transformation method. Positive colonies were then selected and sequenced to verify the correct annotation and nucleotide sequences.

### Induction of multiantigenic recombinant protein

The clone of OvCB_OvAEP_OvCF in pET32a+ in *E. coli* strain BL21(DE3) was cultured overnight (16–18 h) at 37 °C in 50 mL LB broth containing 100 μg/mL of ampicillin with agitation at 200 rpm. The bacterial pellet was collected as a starter for further induction of protein expression in 200 mL fresh LB broth containing 100 μg/mL of ampicillin and 1 mM isopropyl β-D-1-thiogalactopyranoside (IPTG). After induction of the protein expression for 8–10 h, the bacterial pellet was harvested and carried out to break in denaturing binding buffer (5 mM imidazole, 0.5 M NaCl, 20 mM Tris–HCl, 8 M urea, pH 7.9) by freeze–thaw and sonication on ice (4 °C) at 25% amplitude for 5 min with a pulse on and off every 5 s using an ultrasonic sonicator (VCX 750 W, Sonics). The bacterial debris was then removed, and the supernatant was collected for further purification.

The supernatant containing the desired multi-antigenic recombinant protein was purified in denaturing conditions using nickel–nitrilotriacetic acid (Ni–NTA) column (HisPur™ Ni–NTA resin, Thermo Scientific™). Then, the purified protein was analyzed using 15% sodium dodecyl sulphate-polyacrylamide gel electrophoresis (SDS-PAGE) and the protein concentration was measured using NanoDrop Microvolume Spectrophotometer (Thermofisher Scientific). Finally, the purified recombinant protein was aliquot at 1 mg/mL and stored at −20 °C for further analysis.

### Evaluation of the multi-epitope recombinant protein for diagnosing opisthorchiasis using immunoblotting

The multi-epitope recombinant protein was used as the target antigen to establish an immunoblotting assay for the diagnosis of opisthorchiasis. Briefly, 50 μg of recombinant protein was separated on 15% SDS-PAGE at 100 V, 200 mA using Bio-Rad electrophoresis system. Separated proteins were transferred onto a nitrocellulose membrane (0.45 μm, Thermofisher Scientific) using a wet tank transfer system (Cleaver Scientific, United Kingdom) at 50 V and 200 mA for 3 h. The transferred membrane was cut into 5 mm wide strips estimated to contain approximately 5 μg of protein per strip for immunoblotting.

The strips were washed several times with phosphate-buffered saline with Tween 20 (PBST) before blocking non-specific binding sites with PBST-3% BSA in a mosied chamber for overnight at 4 °C. Subsequently, the strips were proceeded to washing steps and carried out to incubated with the sera from patients with parasitic infections at a dilution of 1:250 in PBST-1.5% BSA for 2 h at room temperature (25 °C) with agitation. The strips were then washed and proceeded to incubate with goat anti-human IgG antibody (Rockland) at dilution of 1:2000 in PBST-1.5% BSA for 2 h at room temperature (25 °C) with agitation. After removing the secondary antibodies, the strips were washed with PBST and PBS. The reactivity of the protein bands was detected using TMB-Blotting Substrate Solution (Thermofisher Scientific). The reactivity was stopped by washing with tap water. Positive reactivity was indicated by the blue color of the protein bands. Positive (pool-positive *O. viverrini* infection human sera) and negative controls (pooled non-parasitic infection human sera) were used for quality control immunoblotting and validation of the results.

### Statistical analysis

Immunoblot performance in the diagnosis of opisthorchiasis was evaluated. The performance characteristics of the test, including sensitivity (true-positive rate), specificity (true-negative rate), false-positive rate (FPR), false-negative rate (FNR), positive predictive value (PPV), negative predictive value (NPV), and diagnostic accuracy, were analyzed on the basis of the categorization of the results obtained in a 2 × 2 table [18].

## Results

### Selection of DNA encoded B-cell epitope-rich peptides

OvCB, OvAEP, and OvCF carry several peptide sequences that represent B-cell epitopes (Table 1). Peptide sequences with the highest epitope probability scores were selected. Five epitopes of OvCB included amino acids from 39 to 227, six epitopes of OvAEP included amino acids from 249 to 357, and two epitopes of OvCF included amino acids from 78 to 163 were selected for cloning (Table 1).

### Construction of recombinant DNA

The recombinant OvCB_OvAEP_OvCF_pET32a+ DNA was constructed. The total length of the recombinant OvCB_OvAEP_OvCF DNA in the pET32a+ vector was 1224 nucleotides, and the expected molecular weight of the recombinant protein was 54.7 kDa. The deduced amino acid sequence of recombinant OvCB_OvAEP_OvCF_pET32a+ was constructed, and the order of nucleotides and integration of DNA fragments in pET32a+ were verified via sequencing using T7 sequencing primers (Fig. 1), which revealed several point mutations in the nucleotide sequence of recombinant OvCB_OvAEP_OvCF_pET32a+. However, the mutation was silent and the subsequent amino acid sequence of the protein remained unaffected (Additional file 1: Fig. S1).

### Recombinant protein production and purification

**Fig. 2:**
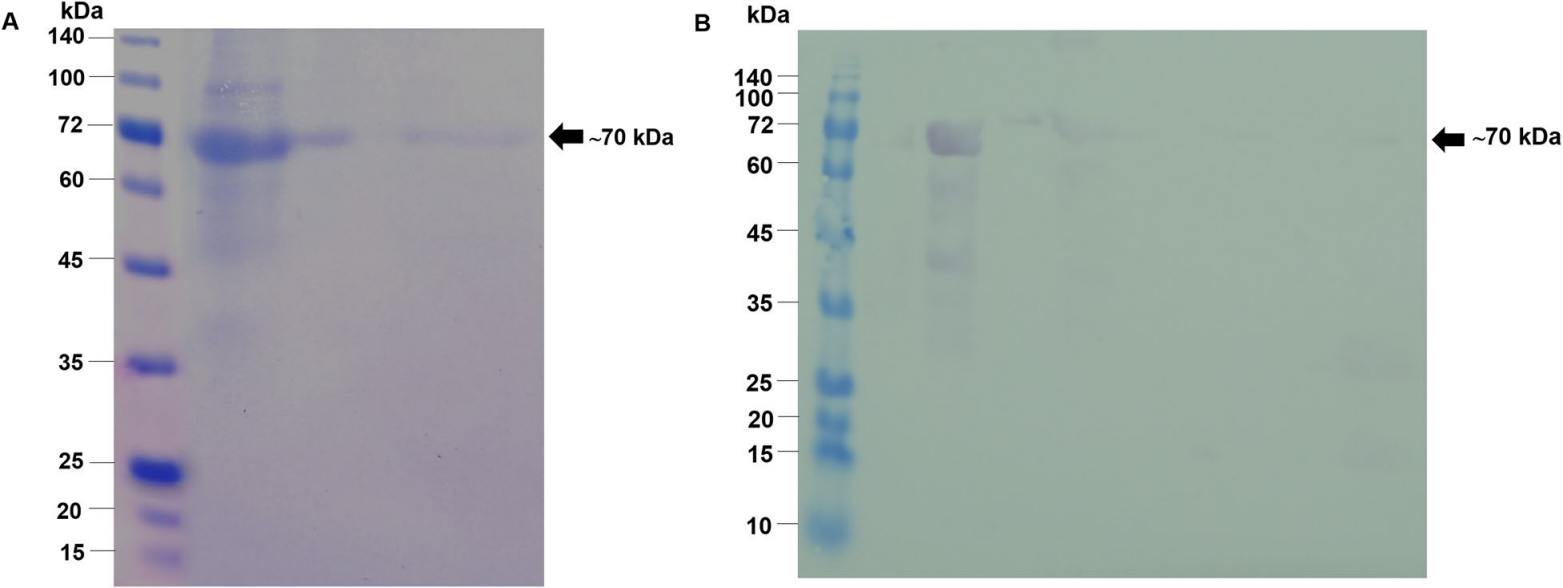
The recombinant OvCB_OvAEP_OvCF protein fused with 6 × His in both N- and C-terminals in pET32a+ system. The recombinant protein expressed in *Escherichia coli*, BL21(DE3), was larger than what was expected. The correspondence of molecular weight of the recombinant protein on 15% SDS-PAGE (**A**) and immunoblot with anti-His tag antibody (**B**) was observed at approximately 70 kDa

The recombinant protein OvCB_OvAEP_OvCF_pET32a+ was produced in *E. coli* BL21(DE3). The recombinant protein with His–tag at the amino- and carboxyl-terminal ends was expressed in an insoluble form. The expressed proteins were dissolved in 8 M urea-binding buffer and partially purified on a Ni–NTA column. However, the high content of acidic amino acids in the protein causes purified recombinant proteins to have a molecular weight larger than their predicted size [19]. Thus, using SDS-PAGE and immunoblotting with an anti-His tag antibody, the molecular weight of the recombinant protein was determined to be approximately 70 kDa (Fig. 2).

### Evaluation of recombinant protein in the diagnosis of opisthorchiasis using immunoblotting

The potential of the recombinant protein OvCB_OvAEP_OvCF_pET32a+ as a diagnostic antigen for opisthorchiasis was evaluated using immunoblotting. The strong immunoreactivity of all opisthorchiasis human sera (35 cases; 100% sensitivity) at the recombinant protein band of approximately 70 kDa was observed. The strong reactivity of opisthorchiasis sera to recombinant proteins was unrelated to the intensity of infection.

The specificity of the recombinant protein was evaluated using 20 serum samples from subjects with other parasitic infections and 17 negative serum samples from subjects with non-parasitic infections in an endemic area. Among samples from subjects with other parasitic infection, the serum IgG antibodies from taeniasis (T) (two cases), strongyloidiasis (SS) (one case), giardiasis (GL) (one case), *E. coli* (EC) infection (two cases), enterobiasis (EV) (one case), and mixed infection of *Echinostome* spp. and *Taenia* spp. (E&T) (one case) were reacted with the recombinant protein. No reactivity was observed in the sera of the subjects with non-parasitic infections (Additional file 3: Fig. S3). The cross-reactivity with other parasitic infections and no reactivity with non-parasitic infections showed 78.4% specificity for detection by immunoblotting. In addition, FNR, FPR, PPV, and NPV were 0%, 21.6%, 81.4%, and 100%, respectively. Moreover, the diagnostic accuracy was 88.9% (Table 2).

**Table 2:** 2 × 2 table represents the performance of immunoblotting in detecting specific IgG antibodies against the recombinant protein OvCB_OvAEP_OvCF_pET32a+

Immunoblotting	FECT		Total
	Positive opisthorchiasis	Negative	
Positive	35 (TP)	8 (FP)	43
Negative	0 (FN)	29 (TN)	29
Total	35	37	72

## Discussion

The shift to light infections necessitates sensitive methods for diagnosing opisthorchiasis in many endemics [1–4]. Antigen–antibody reactivity can be effectively exploited to detect light-intensity *O. viverrini* infection. In addition, using multi-epitope proteins as target antigens can increase the detection sensitivity.

The multi-B-cell epitope protein BSjPGM-BSjRAD23-1-BSj23 is an example of a constructed antigen with potential as a diagnostic target. BSjPGM-BSjRAD23-1-BSj23 showed high sensitivity (97.8%, 89/91) and specificity in diagnosing goat schistosomiasis with reduction of cross-reactivity with goat hemonchosis and orientobilharziasis [17].

In this study, the single immunogenic molecules, OvCB, OvAEP, and OvCF, contained several B-cell epitopes. The combination of OvCB, OvAEP, and OvCF epitopes for B cells contributes to a higher number of antigenic protein targets, which increases the sensitivity of diagnosing opisthorchiasis.

The high levels of antibodies specific to *O. viverrini* infection, particularly total IgG antibody in the serum [20], as well as the immunogenicity of the recombinant OvCB_OvAEP_OvCF protein, provided a sensitivity of 100% and specificity of 78.4% for diagnosing opisthorchiasis. The strong reactivity with total IgG antibodies in the serum from light-infection opisthorchiasis observed in this study allows for efficient screening of light *O. viverrini* infections in both endemic and non-endemic areas. In addition, the high immunogenicity of the OvCB_OvAEP_OvCF protein could promote its detection in chronic and heavy infection cases where immunosuppression has been indicated [21].

Nevertheless, the recombinant OvCB_OvAEP_OvCF protein also showed strong reactivity to total IgG antibodies against other endemic helminth and protozoan infections. The highly conserved OvCB, OvAEP, and OvCF across parasitic helminths and protozoa allow for cross-reactivity with sera from patients with other parasite-infected [14, 16, 22]. Furthermore, IgG antibodies persisted in *O. viverrini*-infected hosts after antihelmintic treatment [23], and patients with a light infection excreting fewer than 50 eggs/g feces, which were undetectable by FECT, showed positive reactivity with the recombinant protein [24]. The detection of IgG4 antibodies [25] or the engineering of recombinant protein targets using rational design and directed evolution can improve the affinity and specificity of protein target binding to specific antibodies, thereby increasing the sensitivity and specificity of diagnosis [26, 27]. Moreover, other immunoglobulin isotypes or specific antibodies should be detected in alternative samples, such as saliva and urine.

Furthermore, the high immunogenicity of the recombinant OvCB_OvAEP_OvCF protein, which promotes high sensitivity in the detection of opisthorchiasis, could facilitate practical screening. The high sensitivity of multi-epitope protein target detection may enable patients with mild infections to be enrolled in confirmatory tests. Thus, OvCB_OvAEP_OvCF may be another target for controlling and preventing opisthorchiasis and CCA.

## Conclusions

Proteins that present epitopes on B cells can stimulate host immune responses and serve as targets for detecting specific host humoral immune responses that diagnose infections. Combining multiple proteins that present B-cell epitopes, including OvCB, OvAEP, and OvCF, could result in a highly immunogenic protein with high sensitivity for the diagnosis of low-intensity *O. viverrini* infections. Although the prevalence of opisthorchiasis has decreased, mild infections continue to be reported and are difficult to diagnose using traditional microscopic methods. Thus, detecting the specific antibody to a single multi-B cell epitope protein, OvCB_OvAEP_OvCF, which demonstrated a high sensitivity of 100% and specificity of 78.4% for diagnosing light-intensity *O. viverrini* infection, was found to be an efficient screening method before proceeding with the confirmation test. The high immunogenicity of the OvCB_OvAEP_OvCF protein suggests that it can be used as a screening target in both endemic and non-endemic areas. Nonetheless, further studies are needed to increase the specificity of OvCB_OvAEP_OvCF detection. Additionally, the development of a screening test capable of detecting different immunoglobulins or infections in different sample types should be considered.

### Supplementary Information

**Additional file 1: Fig. S1.** Nucleotide sequence alignment. The graphic represents the nucleotide sequence alignment of the original OvCB, OvAEP, and OvCF sequences and the recombinant DNA obtained from sequencing. Edited nucleotides in the consensus sequence are indicated by lowercase letters.

**Additional file 2: Fig. S2.** Alignment of OvCB, OvAEP, and OvCF protein sequences with the recombinant protein sequence OvCB_OvAEP_OvCF_pET32a + .

**Additional file 3: Fig. S3.** Immunoblotting with specific IgG antibodies against the recombinant protein OvCB_OvAEP_OvCF_pET32a+. All sera from patients with opisthorchiasis (A and B) showed a band of specific reactivity corresponding to the molecular weight of the recombinant protein. Cross- and false-reactivity were observed in sera with taeniasis (T), *E. coli* infection (EC), enterobiasis (EV), giardiasis (GL), strongyloidiasis (SS), and mixed infection with *Echinostome* spp. and *Taenia* spp. (E&T) (C). The absence of reactivity was observed in sera from negative subjects (D).

## Data Availability

The original data supporting the findings of this study are available within the article and its supplementary materials.

## Abbreviations

PCR: Polymerase chain reaction
dNTP: Deoxyribonucleotide triphosphate
IPTG: Isopropyl β-D-1-thiogalactopyranoside
rpm: Revolutions per minute
SDS-PAGE: Sodium dodecyl sulphate-polyacrylamide gel electrophoresis
PBS: Phosphate–buffered saline
PBST: Phosphate-buffered saline with Tween 20
BSA: Bovine serum albumin
Ni–NTA: Nickel–nitrilotriacetic acid
